# Enhancement Patterns in Differentiated and Undifferentiated Gastric Carcinoma: Multiphasic Contrast-Enhanced Computed Tomography Versus Histopathology

**DOI:** 10.7759/cureus.60841

**Published:** 2024-05-22

**Authors:** Sangeetha S., Rakesh Sankaran, Shaik Farid

**Affiliations:** 1 Radiodiagnosis, Karpaga Vinayaga Institute of Medical Sciences and Research Centre, Chennai, IND

**Keywords:** undifferentiated type, differentiated type, tnm staging, histopathologic correlation, multiphasic contrast-enhanced ct, gastric carcinoma

## Abstract

Background

Gastric adenocarcinoma (GCA) poses a significant global health burden due to its prevalence and high morbidity and mortality rates. GCA is classified into three main histological types: well-differentiated (intestinal type), poorly differentiated (diffuse type), and mixed or indeterminate forms. These types vary in causes, epidemiology, and genetics, with the diffuse type often associated with the worst prognosis.

Endoscopic biopsy is the primary method for characterization, but it has its limitations. There is potential in using contrast-enhanced computed tomography (CT) to differentiate between histological subtypes of gastric adenocarcinoma, which could aid subtype differentiation. Building on this, our study aims to assess CT's effectiveness in distinguishing between broad histological groups of gastric adenocarcinoma based on enhancement patterns, contributing to improved diagnostic accuracy

Objective

Our research focuses on evaluating the effectiveness of multiphasic contrast-enhanced computed tomography (CECT) in distinguishing between the three broad histopathological subtypes of gastrointestinal cancers.

Methods

This study was a prospective, analytical observational study that was approved and carried out in our institutional tertiary care hospital. Consecutive individuals who had undergone endoscopic-guided biopsy and demonstrated histological evidence of GCA were taken into consideration for participation in the study. In order to complete the clinical staging process, further multiphasic CT scans were carried out on each of the fifty patients and were categorised accordingly based on the findings of histopathology.

Results

In the differentiated type, segmental distribution was: 5.5% upper segment, 16.7% middle segment, 66.7% lower segment, and 11.1% diffuse type. Esophageal involvement was 5.6%, duodenal involvement was similar, and lymph node involvement was approximately 38.8%. TNM staging: 38.8% IIIB, 22.2% III, 27.8% IVA, and 11.1% IVB. In the undifferentiated type, segmental distribution: 6.2% upper segment, 31.2% middle segment, 50.0% lower segment, and 12.5% diffuse type. Esophageal involvement was around 6.25%, duodenal involvement was 18.75%, and lymph node involvement was about 71.8%. TNM staging: 34.4% IIIB, 21.8% III, 28.1% IVA, and 15.6% IVB.

Conclusion

Multiphasic CT evaluations provide valuable insights into the prognostic aspects of gastric carcinomas by assessing peak enhancement. Differentiated tumors typically exhibit arterial phase enhancement, while undifferentiated tumors show venous phase enhancement, reflecting their microvascular architecture. Recent studies emphasize the importance of understanding gastric carcinoma characteristics for diagnosis and prognosis. Our research aligns with this, revealing distinct contrast enhancement patterns between differentiated and undifferentiated types. However, discrepancies in histological classifications and contrast enhancement patterns across studies warrant further investigation. Integrating histopathological and radiological insights is essential for accurate diagnosis and treatment planning.

## Introduction

Gastric adenocarcinoma, commonly referred to as GCA, has a global prevalence and carries significant morbidity and mortality. Lauren et al. classified them primarily into three types based on histology: well-differentiated type also known as intestinal type, poorly differentiated type also known as diffuse, and mixed forms or indeterminate type [1]. The types of these tumours differ in the aspects of their causes, epidemiology and genetics [2,3]. Their characterization plays an utmost important role in determining their prognosis, with the worst clinical course often associated with diffuse type.

Yasuda et al. found that overall survival rate and prognosis were good with well-differentiated gastric adenocarcinoma (76%) compared to the poorly differentiated type (67%), particularly among patients with tumors thickness measuring greater than 10 centimeters or more (42 % versus 14 %; p = 0.017) [4]. Currently, endoscopic-guided biopsy is the only method for histological characterization of GCA. However, endoscopy has limitations, including the potential for the collected sample to inaccurately represent the entire tumor, which can exhibit geographical and histological variations [5].

Furthermore, endoscopy cannot determine the tumor extent beyond the intramuscular space or identify distant nodal and visceral metastases. Previous research using computed tomography (CT) aimed to differentiate between various histological forms of genetically modified cancer [6-12]. Tsurumaru et al. explored the utility of contrast-enhanced multidetector CT gastrography (CE-CTG) to assess peak enhancement in GCAs [10]. By analysing their enhancement pattern on contrast-enhanced CT scans, predictions regarding the three main histopathological subtypes of gastric adenocarcinoma are possible.

In line with this, our research focuses on evaluating the effectiveness of contrast-enhanced computed tomography in distinguishing between the three broad histological groups of gastrointestinal cancers.

## Materials and methods

This study was a prospective, analytical observational study that was approved and carried out in our institutional tertiary care hospital. The patients who had undergone endoscopic guided biopsy by direct visualisation and had histopathological evidence of gastric carcinoma were taken into consideration for participation in the study. An upper gastrointestinal endoscopy was performed for all the patients within the study group with the help of a Pentax gastroscope (Pentax Medical India Private Limited, Gurgaon, India). In order to complete the clinical staging process, further multiphasic CT scans were carried out on each of the fifty patients and were categorised based on the findings of histology.

The following categories were used to classify the tumours that we found during the course of our research: Among the three categories, the first one is differentiated, the second one is undifferentiated, and the third one is mixed. The preceding classification was arrived at by giving consideration to the criteria that are presented in the following list. Well-differentiated, moderately differentiated and papillary adenocarcinomas were included in the category of differentiated cancers. Undifferentiated types of gastric carcinoma included poorly differentiated adenocarcinomas, signet-ring cell type of adenocarcinoma carcinomas, and mucinous variety. People were regarded to be of mixed type if they exhibited characteristics of both differentiated and undifferentiated.

All 50 patients underwent a multiphasic (arterial, portal-venous and venous) CT scan, which was carried out with the assistance of a 128-slice multidetector scanner. The patient was required to abstain from food and drink for the whole night as part of the preparation process. Immediately prior to the examination that was going to take place in the CT suite, a negative contrast consisting of between 600 and 1000 millilitres of water was administered to everyone individually. This was done in order to generate a sufficient amount of distension in the stomach. Study group patients did not receive any form of anti-peristaltic medicine to treat their condition. Obtaining pre-contrast axial photos of the upper abdomen required the use of the following settings, which were utilised: By default, the parameters for the slice thickness and intervals are set to 5 millimetres. A power injector was utilised to administer intravenously a non-ionic contrast medium at a rate of 3-4 ml/s. In order to carry out a triphasic post-contrast CT scan, it was carried out subsequently. During the arterial phase at 20-30 seconds, images of the upper abdomen in axial cuts were obtained. The entire abdomen was imaged during the portal-venous phase at 30-40 seconds and then the upper abdomen cuts were obtained once more during the venous phase at 50-55 seconds. These times are determined after the intravenous injection has been started. For this particular experiment, the timings that were used were exactly the same as was anticipated. Both the pre and post-contrast scans were fairly similar in acquisition parameters to one another in terms of their similarities and differences.

In order to determine whether or not lung metastases were present, axial sections of the chest were obtained during both the pre- and post-contrast phases; however, these images were not included in the research. Each and every axial acquisition was reconstructed to a thickness of one millimetre for each and every axial slice. Following the completion of the axial reconstructions of 1 millimetre for each of the three phases, the reformations of the coronal and sagittal sections were reconstructed. The CT dataset was analysed by two qualified radiologists who are experts in abdominal radiology, and their interpretations of the pictures were supplied. In spite of the fact that neither of the radiologists was informed of the histology findings, they did make use of the endoscopic findings as a point of reference.

**Figure 1:**
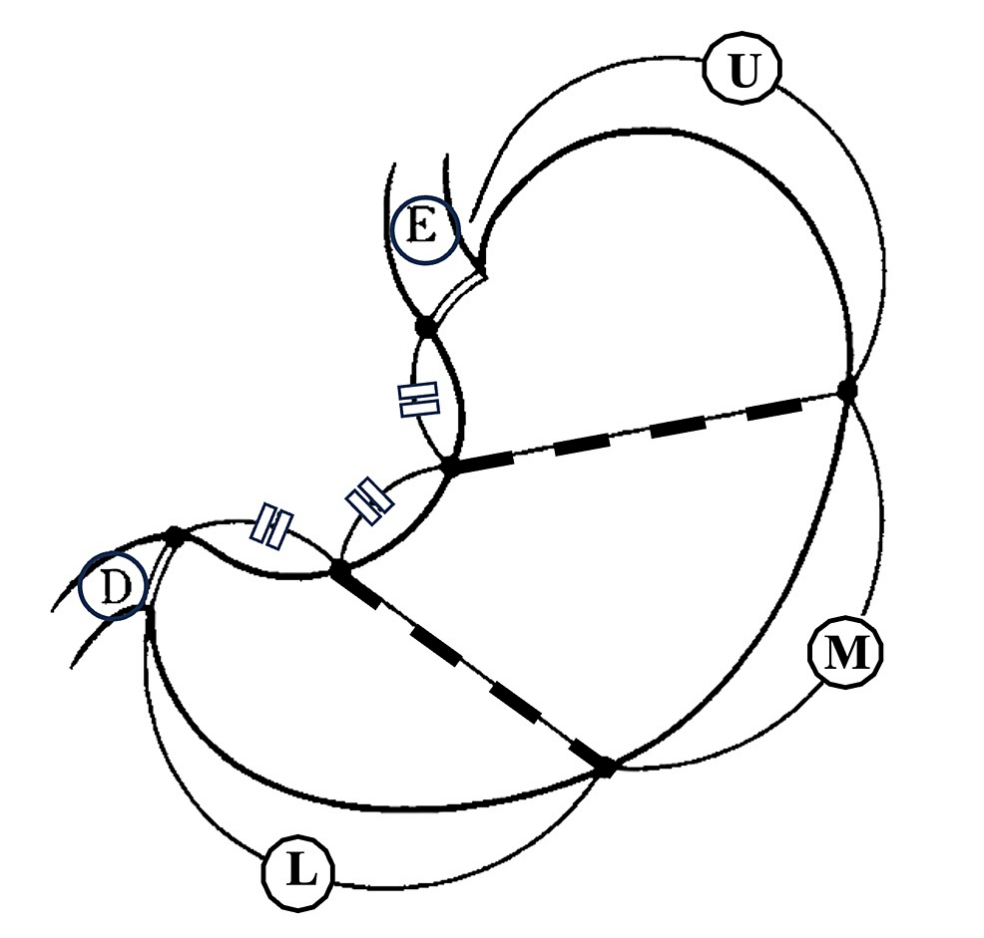
The three sections of stomach Upper third (U), Middle third (M), Lower third (L) formed by the line connecting the trisected points on the lesser and greater curvatures, Esophagus (E), Duodenum (D)

Observations were made according to the following. The location of the tumour is as follows: A physical separation of the stomach into three distinct sections the upper (U), middle (M), and lower (L) parts were used for the purpose of this analysis. It is done by drawing a line connecting the trisected points on the lesser and greater curvatures as shown in Figure 1 [12]. If more than one segment of the stomach was involved, the lesion was classified according to the region that has the major tumoral involvement e.g., LM or UML. This was done in cases when more than one segment was involved. In the event that all three segments were damaged by the tumour, it was generally accepted that a diffuse tumour was present (D). Furthermore, extensions into the oesophageal and duodenum were noted as ES and DU, which are the regions that are pertinent to the discussion. There are a number of variables that are linked to the enhancement of the tumour peak: In addition to the pre-contrast phase images, it was the responsibility of the readers to manually delineate areas of interest (ROIs) at the maximum Hounsfield value (HU) for each of the post-contrast studies. It was decided to just take the average of the greatest values (HU) for the purpose of the potential readers. The enhancement phases of the lesions were separated into three categories: arterial, portal-venous, and venous phase. These categories were determined by the phase that had the maximum attenuation value.

The eighth tumour staging Tumor Node Metastasis (TNM) system developed by the American Joint Committee on Cancer (AJCC) was utilised in order to accomplish the desired staging [13].

The Chi-square test was carried out to determine whether there was a difference in enhancement patterns of differentiated and undifferentiated gastric carcinomas. Samples were obtained through independent sampling. With the help of a T-test, we were able to determine whether or not the contrast research was able to discern between the two types of GCA in an appropriate manner. An analysis of the tumour, lymph nodes, and metastases is performed.

## Results

**Figure 2:**
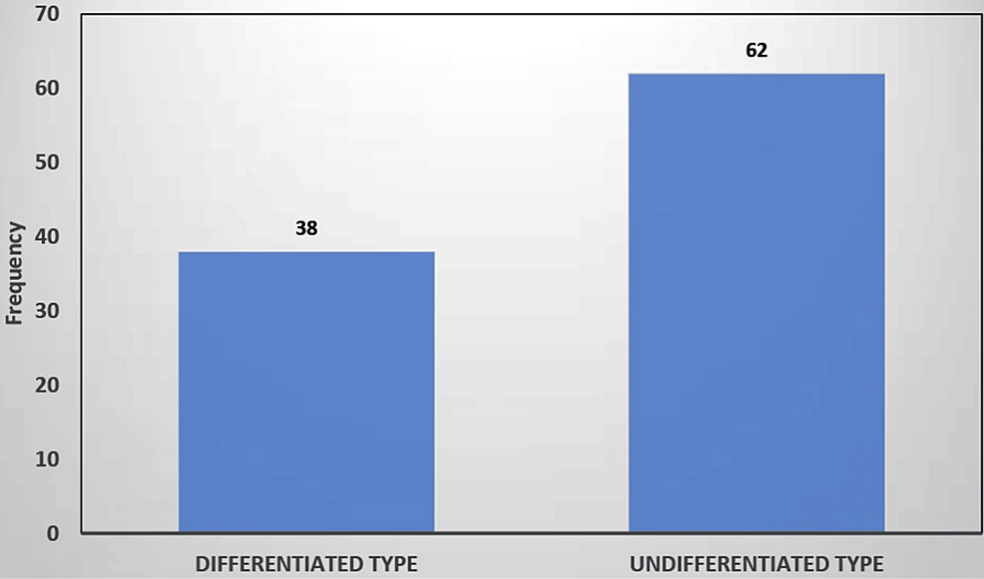
Distribution of histological subtype among the study participants (N=50)

In our study, around 38% had histopathological differentiated type and 62% undifferentiated type (Figure 2).

In the differentiated type, 5.5% were situated in the upper segment, 16.7% in the middle segment, 66.7% in the lower segment, and 11.1% exhibited a diffuse type. Approximately 5.6% showed involvement in the oesophagus, and a similar percentage had duodenal involvement. Lymph node involvement was observed in around 38.8% of cases. Regarding TNM staging, 38.8% were classified as IIIB, 22.2% as III, 27.8% as IVA, and 11.1% as IVB (Table 1).

**Table 1:** Distribution of histopathological differentiated type, location, extent, and tumour staging with gastric adenocarcinoma (n = 18)

Slno	Variable	Frequency	Percentage
1	Location		
	Upper	1	5.5
	Middle	3	16.7
	Lower	12	66.7
	Diffuse	2	11.1
2	Oesophageal involvement	1	5.6
3	Duodenal (DU) involvement	1	5.6
3	Lymph nodal involvement	7	38.8
4	Hepatic metastasis	1	5.6
5	TNM stage		
	≤ IIA	0	0
	IIB	7	38.8
	III	4	22.2
	IVA	5	27.8
	IVB	2	11.1

In the undifferentiated type, 6.2% were situated in the upper segment, 31.2% in the middle segment, 50.0% in the lower segment, and 12.5% exhibited a diffuse type. Approximately 6.25% showed involvement in the oesophagus, and 18.75% percentage had duodenal involvement. Lymph node involvement was observed in around 71.8% of cases. Regarding TNM staging, 34.4% were classified as IIIB, 21.8% as III, 28.1% as IVA, and 15.6% as IVB (Table 2).

**Table 2:** Distribution of histopathological undifferentiated type, location, extent, and tumour staging with gastric adenocarcinoma (n = 32)

Slno	Variable	Frequency	Percentage
1	Predominant gastric segment location of the tumour		
	Upper	2	6.2
	Middle	10	31.2
	Lower	16	50.0
	Diffuse	4	12.5
2	Oesophageal involvement	2	6.25
3	Duodenal (DU) involvement	6	18.75
3	Lymph nodal involvement	23	71.8
4	Hepatic metastasis	5	15.6
5	TNM stage		
	≤ IIA	0	0
	IIB	11	34.4
	III	7	21.8
	IVA	9	28.1
	IVB	5	15.6

The peak-enhancement type was ‘arterial’ in 12 out of 18 within the differentiated-type GCAs and ‘portal-venous’ in 7 out of 25 within the undifferentiated-type GCAs (the chi-square statistic with Yates correction is 8.001. The p-value is .004675) (Table 3).

**Table 3:** Association of enhancement pattern on contrast-enhanced computed tomography for gastric adenocarcinomas (N=50)

Slno	Histopathological type	Enhancement phase		p-value
		Arterial	Portal-venous	
1	Differentiated Gastric type	12	6	p-value : 0.004675 (Yates correction - 8.001)
2	Undifferentiated Gastric type	7	25	

The average maximum attenuation values (in Hounsfield Units, HU) for 50 cases of gastric carcinoma (GCA) on multiphasic CT are presented in Table 4. In the arterial phase, the mean maximum attenuation value was higher for differentiated GCAs, whereas in the portal-venous phase, it was higher for undifferentiated GCAs. A statistically significant difference in maximum attenuation values was found between differentiated and undifferentiated GCAs during the arterial phase (p ≤ 0.001). However, there was no statistical significance observed for the portal-venous phase (p = 0.179) and hepatic-venous phase (p ≤ 0.554).

**Table 4:** Association of enhancement pattern on various phases of computed tomography for gastric adenocarcinomas (N=50)

Contrast Phases	Differentiated gastric type	Undifferentiated gastric type	Total	t-value	p-value
Pre-contrast	39.2±6.1	36.80±6.1	38±6.10	1.1169	0.250
Arterial Phase	69.8±10.5	54.70±11.2	62.25±10.85	4.179	0.001
Porto-Venous Phase	65.01±11.9	70.1±11.9	67.55±11.90	-1.363	0.179
Venous phase	50.50±12.01	48.2±11.1	49.35±11.55	0.596	0.554

In our study, we found no substantial statistical variance in lymph nodal involvement and hepatic metastasis between the differentiated and undifferentiated carcinomas. The Chi-square statistic with Yates’s correction yielded a value of 1.751 and a corresponding p-value of 0.176 for lymph node metastasis, while for hepatic metastasis, the Chi-square statistic with Yates’s correction was 0.0078, with a p-value of 0.950.

## Discussion

**Figure 3:**
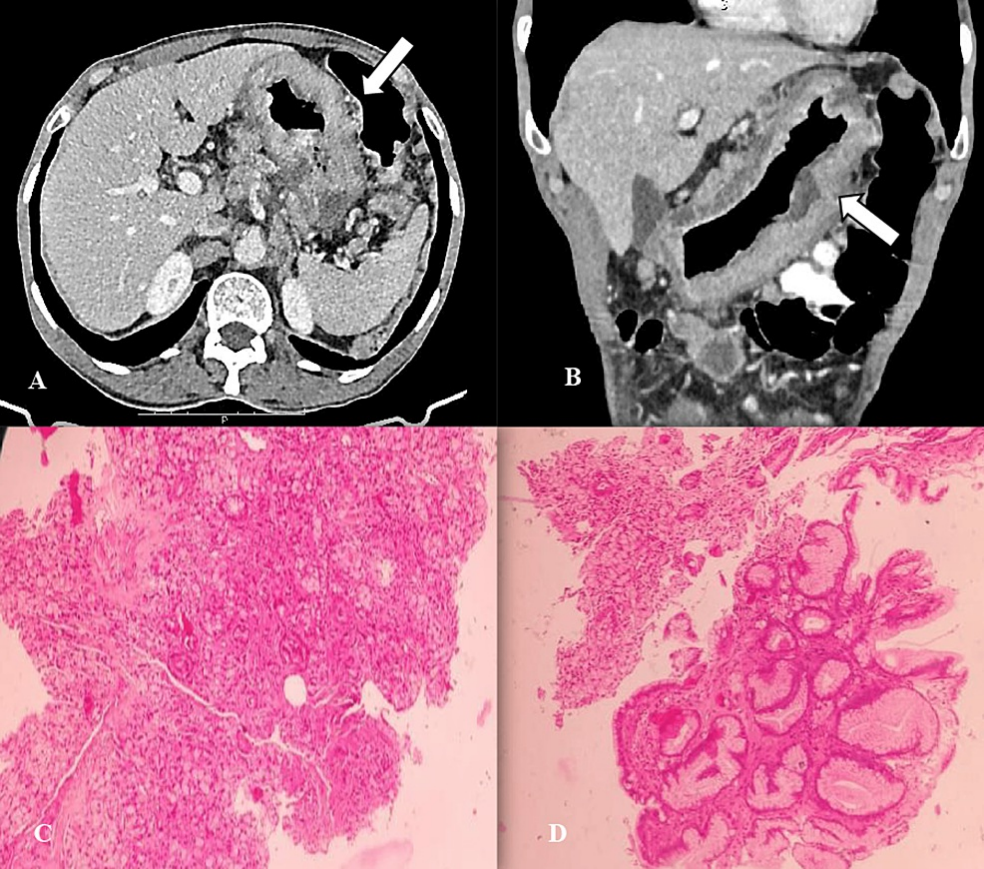
Gastric adenocarcinoma (differentiated type) in a 72-year-old man (A, B) Contrast-enhanced CT shows a diffuse gastric wall thickening indicated by arrows. The enhanced value peaks in the arterial phase, and the Hounsfield values were 75 HU and 59 HU in the portal venous phase. Photomicrograph sections (C, D) of the specimen show well-differentiated gastric adenocarcinoma.

**Figure 4:**
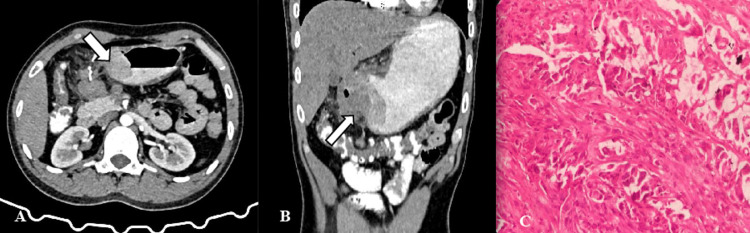
Contrast-enhanced CT [A,B] of a gastric cancer (undifferentiated type) in a 50-year-old woman demonstrates a 5.1 × 3.5 cm mass lesion in the antro-pyloric region indicated by arrows. The contrast enhancement peaked in the portal venous phase, and the attenuation values in the arterial and the porto-venous phases were 54 HU and 90 HU, respectively. (C) Photomicrograph section of the lesion shows undifferentiated gastric adenocarcinoma.

Our study highlights the distribution of gastric adenocarcinoma (GCA) histopathological subtypes, revealing 38% as differentiated (Figure 3) and 62% as undifferentiated (Figure 4). While differentiated cases predominantly exhibited segmental distribution in the lower segment, undifferentiated cases showed a more dispersed distribution across upper, middle, and lower segments. Notably, a proportion of undifferentiated cases presented with a diffuse type.

During their research, Yasuda and colleagues explored that the microvascular architecture of Gastric carcinoma, specifically focusing on histopathological subgroups [4]**.** Differentiated carcinomas were normo-vascular (**65%**) or hyper-vascular (24%), while undifferentiated carcinomas were predominantly hypo-vascular, consistent with histology (60%). Stromal hypervascularity explains the tendency to metastasize to the liver and lungs in well-differentiated gastric carcinoma. However, our study reflects no statistically significant association in the incidence of hepatic metastasis between differentiated and undifferentiated types.

Recent studies have emphasized the growing importance of GCA characteristics for diagnosis, staging, and prognosis. Poorly categorized GCAs with uncertain subtypes often have a poor prognosis. Predicting histology based on CT characteristics could have valuable implications for clinical research and practice. In our study, we found that in patients with the differentiated type, 66.7% exhibited an arterial peak enhancement pattern, while 33.3% showed a portal-venous peak enhancement pattern. Conversely, in patients with the undifferentiated type of carcinoma, 21.8% displayed an arterial peak enhancement pattern, while 78.1% had a portal-venous peak enhancement pattern.

In a study by Wankhar et al [14] and Kawamura et al [15], there was a significant difference in the enhanced pattern between differentiated gastric and undifferentiated gastric adenocarcinoma, with a statistically significant distinction. Differentiated GCAs predominantly exhibited arterial phase dominance enhancement (71.4%), while undifferentiated GCAs showed portal-phase dominance enhancement (88%), aligning with Yasuda et al.'s [4] of microvascular architecture influence.

Tsurumaru and colleagues [10] conducted a similar study in the past, utilizing multiphasic CT for a retrospective analysis of advanced GCA patients. They reported greater Hounsfield values in undifferentiated gastric carcinoma compared to mixed and differentiated subtypes of GCTs during the delayed phase. In contrast to our findings, they suggested no substantial change in the arterial phase between histological classifications. However, direct comparison is challenging due to alterations in the CT process.

Tsurumaru et al [10] also examined diffuse-type advanced GCAs using contrast-enhanced CT, revealing a two-layered pattern during the arterial phase, contrasting our observations. Lee et al. investigated contrast enhancement in gastric cancer, finding a higher incidence of signet ring cell carcinoma (SRC) during the portal-venous phase, attributed to SRC's immature fibrosis symptoms.

In our research, there were no statistically significant differences in peak enhancement patterns in the venous phase between diffuse gastric carcinoma with signet ring histology and other cases.

Limitations

Our study had its own limitations: it was a single-centered institutional study with a relatively modest sample size. However, considering it is a prominent tertiary care facility in the area, these results could carry notable clinical implications. The power of the study was by post-hoc power is 90% which implies the study was adequately powered to detect the observed effect size, suggesting confidence in the study's findings. The study's findings could have been affected by sampling bias, as it exclusively enrolled patients from a single tertiary care center, possibly limiting its relevance to the wider population. Conducting multicentered research could address this limitation effectively. Furthermore, measurement bias may have been present due to discrepancies in interpreting contrast-enhanced computed tomography (CECT) scans or histopathological assessments. However, this potential bias was mitigated through the implementation of standardized protocols and blinding techniques. The study primarily focused on major histological categories and assessed the maximum attenuation value (HU) within types of gastric carcinoma. This may not fully represent tumor heterogeneity. Additionally, our CT protocol's phase definitions differed from previous studies, complicating direct comparisons. Despite these limitations, our study provides valuable insights into GCA microvascular architecture.

## Conclusions

Our findings underscore the heterogeneity of GCA, both in terms of histopathology and clinical features. Understanding these variations is crucial for accurate diagnosis, staging, and treatment planning, emphasizing the importance of a comprehensive approach in managing patients with gastric adenocarcinoma.

The evaluation of the peak enhancement that occurs during a multiphasic CT examination might offer useful information regarding the prognostic aspects of gastric carcinomas by providing insights into the histological subtypes of these forms of cancer. It is common for differentiated carcinomas to have peak enhancement during the arterial phase, whereas undifferentiated carcinomas typically exhibit peak enhancement during the venous phase. This is likely a reflection of their microvascular architecture, which is in line with the findings of earlier investigations.
